# Protection of Chinese painted quails (*Coturnix chinensis*) against a highly pathogenic H5N1 avian influenza virus strain after vaccination

**DOI:** 10.1007/s00705-013-1754-z

**Published:** 2013-06-15

**Authors:** Julia Sarkadi, Mate Jankovics, Zoltan Kis, Jozsef Skare, Kinga Fodor, Eva Gonczol, Ildiko Visontai, Zoltan Vajo, Istvan Jankovics

**Affiliations:** 1 Division of Virology, National Center for Epidemiology, Budapest, 1097 Hungary; 2 Medical and Health Science Center, University of Debrecen, Debrecen, 4032 Hungary

**Keywords:** Chinese painted quails, HPAI H5N1 strains, NIBRG-14 vaccine, A/Swan/Nagybaracska/01/06(H5N1) challenge virus

## Abstract

Chinese painted quails immunized with a single dose (6 μg HA) of inactivated H5N1 (clade 1) influenza vaccine NIBRG-14 and challenged with 100 LD_50_ of the heterologous A/Swan/Nagybaracska/01/06(H5N1) (clade 2.2) strain were protected, whereas unvaccinated quails died after challenge. No viral antigens or RNA were detected in cloacal swabs from immunized animals. Sera obtained post-immunization gave low titres in serological assays against the vaccine and the challenge viruses. Our results demonstrate the protective efficacy of the NIBRG-14 strain against the challenge virus and the usefulness of these small birds in protection studies of influenza vaccines.

Highly pathogenic avian influenza virus (HPAI) strains cause significant mortality in unvaccinated chickens, but their virulence varies in mammals, and mutations in certain viral genes coding for structural and non-structural proteins contribute to virulence [7, 14, 18]. HPAI strains also cause human infections with high mortality rates, some with a high probability of human-to-human transmission, and they are thus a constant threat to humans [27].

When the mammalian virulence of HPAI H5N1 strains, including human isolates, was tested either in mice or in ferrets, these strains proved to be highly pathogenic or caused only mild or no symptoms. Few studies have measured virulence in both mice and ferrets; some strains have displayed similar pathogenicity in both animals, but other HPAI strains have exhibited virulence differences, especially at high doses [4, 18, 21, 33]. Inbred mouse strains vary considerably in their ability to resist the H5N1 virus, and the distinct expression profiles of inflammatory mediators have been suggested to control disease severity [1]. Such observations indicate that results obtained in H5N1 mammalian protection models can vary with the animal species and the dose and type of the experimentally applied challenge virus.

The immunogenicity and protection-conferring ability of a candidate vaccine virus, NIBRG-14 (clade 1), have been evaluated in mammalian model systems. The protective effect of the anti-haemagglutinin (HA) and anti-neuraminidase (NA) antibodies in BALB/c mice was shown by injecting the serum into severe combined immunodeficiency mice, which were then challenged with a homologous highly pathogenic H5N1 virus strain [25]. Immunization with the adjuvanted NIBRG-14 vaccine strain conferred protection in mice or monkeys against a lethal challenge with homologous or heterologous strains [9, 10, 22]. In humans, one injection of the NIBRG-14 vaccine triggered antibody responses that cross-reacted with clade 2 H5N1 strains, including the A/Swan/Nagybaracska/01/06(H5N1) reassortant strain, in *in vitro* serological assays [6]. These earlier results demonstrated the cross-clade efficacy of the NIBRG-14 vaccine strain.

Animal model systems that met the requirements of high susceptibility to a great variety of H5N1 viruses, easy animal handling, and fast, cost-effective and reliable results are still needed. The HPAI H5N1 strains tested so far were all pathogenic in chickens [8, 17] and Japanese quails (*Coturnix-japonica*) [11, 13, 19, 32]. The Chinese painted quail (*Coturnix-chinensis*, formerly *Excalfactoria* *chinensis*, in the family Phasianidiae of the order Galliformes) is the smallest “true quail”, common worldwide in aviculture; the average weight of the adult male and female is about 48 and 56 g, respectively. Chinese painted quails are superior to chickens and Japanese quails due to their ease of handling and general care, hardiness, excellent reproductive performance, and less expensive maintenance [26]. Importantly, Chinese painted quails are smaller in size than chickens and Japanese quails. Using Chinese painted quails for vaccine studies with HPAI H5N1 viruses that require enhanced biosafety level 3 (BSL-3+) conditions provides a reasonable opportunity for the inclusion of the birds in appropriate numbers and groups in the experiments, thus achieving scientifically acceptable results. Quails carry sialic acid receptors with the potential of binding of avian and human influenza viruses, thereby serving as an intermediate host for the zoonotic transmission of influenza viruses [30] and potentially as a challenge system for human and avian influenza virus vaccines. We are not aware of published results on the use of Chinese painted quails in influenza vaccine research.

We set out to investigate the possible usefulness of the Chinese painted quail in H5N1 vaccine studies. We demonstrated the high pathogenicity of the wild-type A/Swan/Nagybaracska/01/06(H5N1) (clade 2.2) strain in Chinese painted quails and the protective effect of vaccination with the NIBRG-14 strain (clade 1) against this heterologous strain, demonstrating for the first time that Chinese painted quails are useful, reliable, and economically and technically tractable for measuring the protective effect of vaccination against H5N1 viruses.

In our experiments, 10-week-old Chinese painted quails that were seronegative for currently circulating influenza A and B viruses (K.A.G. Technologies Ltd, Hungary) were immunized with the NIBRG-14 H5N1 (clade 1) strain and challenged with the A/Swan/Nagybaracska/01/06(H5N1) (clade 2.2) strain at week 3 or 6 after immunization.

The NIBRG-14 virus strain, obtained from the National Institute for Biological Standards and Control, London, UK, is a reverse-genetics-derived 2:6 reassortant and one of the available and proposed candidate A (H5N1) vaccines [5]. The seed virus was grown in eggs. The formaldehyde-inactivated whole-virus vaccine, produced by Omninvest Ltd., Hungary, contained 6 μg HA and 0.3 mg aluminum phosphate adjuvant/dose. The preparation and immunogenicity of the vaccine in humans were described earlier [6, 28, 29].

The HPAI challenge strain, influenza A/Swan/Nagybaracska/01/06(H5N1), a wild-type avian influenza virus, was isolated in Hungary and shown to belong to clade 2.2 on the basis of HA sequences [6]. The TCID_50_ of the virus was determined by titration in Madin-Darby canine kidney cells. To determine the LD_50_, birds were inoculated subcutaneously (s.c.) with serial dilutions of the virus in a volume of 100 μl, 4 birds for each dilution, and monitored daily for morbidity and mortality for 2 weeks. The TCID_50_ and LD_50_ titres were calculated by the method of Reed and Muench [23] and compared; 50 TCID_50_ were required to give one LD_50_ in Chinese painted quails.

The immunization and challenge experiments were conducted in the BSL-3+ containment facility at the National Center for Epidemiology, as approved for such use by the Chief Medical Officer of Hungary. The facility was secured by procedures recognized as appropriate by the institutional biosafety officers, facility management and Laboratory Animal Care Committee as well as Hungarian government inspectors. The Chinese painted quails were housed in individually ventilated cages used conventionally for mouse experiments (4 birds/cage).

In the first experimental series, 12 birds were immunized s.c. twice at an interval of 3 weeks with the NIBRG-14 strain in a volume of 100 μl and challenged s.c. in 3 groups of 4 animals at week 6 with 1 or 10 LD_50_ of the A/Swan/Nagybaracska/01/06(H5N1) virus in a volume of 100 μl. Three of the 4 unimmunized control birds that received 1 LD_50_ and all four of the unimmunized animals that received 10 LD_50_ of the challenge virus died within 8 days post-challenge. All of the immunized birds survived and remained healthy, demonstrating the protective effect of the twice-inoculated vaccine virus.

In the second experimental series, 24 birds were immunized with a single dose of the vaccine strain. Three weeks later, 8 birds were bled for serologic testing, and 16, in 2 groups of 8 animals, were challenged with 10 or 100 LD_50_ of strain A/Swan/Nagybaracska/01/06(H5N1). Sixteen unimmunized quails, 8 per group, served as controls and were challenged similarly. All birds in the unvaccinated control groups died within 8 days post-challenge, while all vaccinated quails remained healthy and showed no clinical signs of illness (Fig. 1). The unvaccinated group challenged with the 10 or 100 LD_50_ dose displayed depression, reluctance, decreases in food and water consumption, and increased respiratory rates at 24 h post-challenge. At 48 h and 96 h, the clinical signs were similar, but the weakness and drowsiness were more pronounced, and diarrhoea occurred.

**Fig. 1:**
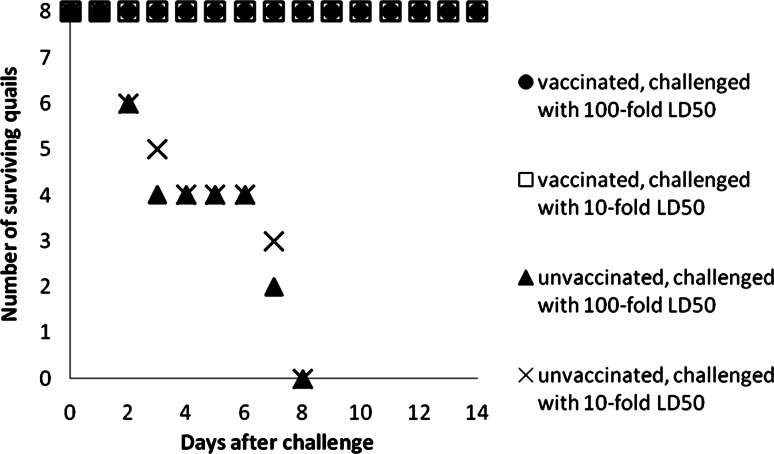
Protection of Chinese painted quails immunized with the NIBRG-14 (clade 1) vaccine against the highly pathogenic heterologous A/Swan/Nagybaracska/01/06(H5N1) (clade 2.2) strain. Sixteen birds vaccinated s.c. with a single dose of NIBRG-14 vaccine (6 HA/dose) were challenged in two groups of eight birds with 10 or 100 times the LD_50_ of the A/Swan/Nagybaracska/01/06(H5N1) strain at 3 weeks post-immunization. Sixteen unvaccinated birds were similarly challenged. Mortality was monitored on a daily basis for 14 days

Cloacal swab samples from immunized and unimmunized animals were analysed for the presence of viral antigens and RNA. The RapidSignal^TM^ Influenza H5 Dipstrip (Orgenics Ltd., Israel) chromatographic immunoassay was used to detect H5 virus antigens according to the instructions of the manufacturer. RNA was extracted with Tri-Reagent (Sigma-Aldrich, St. Louis, MO, USA), and reverse transcription was performed with avian transcriptase according to the manufacturer’s instructions (Applied Biosystems Foster City, CA, USA). REDTaq ReadyMix PCR Reaction Mix with MgCl_2_ (Sigma-Aldrich) was used in the PCR with a primer pair corresponding to the M segment; positive results were confirmed with specific H5 primers [31]. The results showed that H5 viral antigens were present in the cloacal swabs of all the unvaccinated control birds. Those unvaccinated and challenged with 100 LD_50_ were PCR positive, demonstrating the presence of viral RNA in the samples. All samples obtained from immunized and challenged animals were negative for H5 viral antigens and RNA (Table 1). Oropharingeal swabs were not taken from the birds for analysis of viral antigens and RNA.

**Table 1:** Protection from virus excretion in the intestinal tract after challenge of Chinese painted quails immunized with a single dose of the NIBRG-14 strain

Quails challenged with A/Swan/Nagybaracska/01/06(H5N1)	Viral antigens no. of + samples/total no of samples/no. of quails	Viral RNA no. of + samples/total no. of samples/no. of quails
Immunized and challenged with 100 LD_50_	0/40/8	0/40/8
Immunized and challenged with 10 LD_50_	0/40/8	0/40/8
Non-immunized and challenged with 100 LD_50_	14/14/6	14/14/6
Non-immunized and challenged with 10 LD_50_	16/16/7	ND

Quails were immunized with a single dose of the NIBRG-14 vaccine and challenged with the A/Swan/Nagybaracska/01/06(H5N1) strain. Cloacal swab samples were collected on days 0, 2, 4, 6, 8 and 10, but samples were not taken from dead animals. Thirty-two samples from the four groups obtained on day 0 were all negative for viral antigen and RNA (not shown). Results obtained from samples collected on days 2–10 are included in the table

*ND* not done

Sera from 8 birds at 3 weeks after single-dose immunization were tested for anti-HA and neutralization antibodies against the vaccine and challenge strains. Haemagglutination inhibition (HI) tests were performed by standard procedures with chicken red blood cells and 4 HA units of virus/well [16]. The microneutralization (MN) assay was performed as described [31]. Positive antibody controls for HI and MN assays were prepared by immunization of birds with s.c. injection of an emulsion containing 0.5 mg of whole, formaldehyde-inactivated influenza NIBRG-14 four times with complete or incomplete Freund’s adjuvant (Calbiochem, La Jolla, CA, USA). The titre of the positive antibodies against the NIBRG-14 strain was >1:40 in the HI and MN assays. Sera obtained from non-immunized birds did not react in these assays. Statistical analysis was performed with the SPSS program, version 17.0. Differences between groups in continuous variables were calculated using the nonparametric Mann-Whitney U-test; p < 0.05 was taken as significant.

All birds responded with an HI titre of 10.6-32, geometric mean titre (GMT) of 21.1 against the NIBRG-14 homologous strain, but the HI titres against the A/Swan/Nagybaracska/01/06(H5N1) strain were significantly lower, some < 1:4 (GMT = 4.4). When the same sera were examined by MN, the animals gave a GMT of 20.7 against the vaccine strain, and the five animals that exhibited seroconversion against the A/Swan/Nagybaracska/01/06(H5N1) strain in the HI test were also positive in the MN assay. The sera from three birds with a titre of < 1:4 against the challenge virus in the HI tests showed similarly undetectable titres in the MN assays (Table 2). Positive control antibodies exhibited the expected HI and MN titres against the NIBRG-14 strain; negative antibody controls did not react in the assays (not shown).

**Table 2:** Serum antibody responses against the homologous NIBRG-14 and heterologous A/Swan/Nagybaracska/01/06(H5N1) strains in Chinese painted quails after immunization with a single dose of the NIBRG-14 vaccine

Quail no.	Serum antibody titre against			
	NIBRG-14		A/Swan/Nagybaracska/01/06(H5N1)	
	HI	MN	HI	MN
1	16	13.3	8	4
2	32	26.6	10.6	10.6
3	32	32	6.6	8
4	10.6	10.6	4	4
5	16	21.3	<4	<4
6	21.3	26.6	<4	<4
7	21.3	32	<4	<4
8	32	16	8	8
GMT	21.1*	20.7**	4.4*	4.1**

Serum samples from 8 of the 24 immunized quails were collected 3 weeks after a single dose of vaccine. The titres were determined against the homologous and heterologous viruses. Each dilution of the sera was tested in three parallel HI and MN assays, and GMTs were calculated from the data from eight quails. For titres < 1:4, an arbitrary value of 1:2 was used for calculation. *p = 0.001, **p = 0.001

Correlates for protection from disease and death by H5N1 isolates are complex. HI titres of ≥ 1:40 in >70 % of adult subjects are required for the licensing of currently available influenza vaccines [3]. Our results, confirming earlier results [2, 20, 25], suggest that these requirements may not be critical for avian H5N1 viruses and indicate that the low levels or insufficient detection of anti-HA antibodies following immunization with the NIBRG-14 vaccine are not limited to mammals, but are also observed with Chinese painted quail, an avian species that is highly susceptible to a HPAI H5N1 strain. Protection from a lethal viral challenge may possibly be independent of the prechallenge anti-HA antibody levels, and other mechanisms, such as anti-NA antibodies, are involved in protection [24, 25]. Alternatively, anti-HA antibodies were present at a seroprotective level in the sera, but the HI and MN assays detected avian H5N1 antibody with low sensitivity, as suggested earlier [20, 25]. Similarly to our results, cross-reacting antibodies against A/Swan/Nagybaracska/01/06(H5N1) were detected in *in vitro* assays in only about 25 % of humans who were reactive to the homologous NIBRG-1 strain [6]. Furthermore, the vaccine and challenge viruses possessed antigenically distinct HA and NA genes from the clade 1 A/Vietnam/1194/2004 and clade 2 A/Swan/Nagybaracska/01/06(H5N1) strains, respectively, but the cell-mediated immune responses directed to the internal proteins of the strains, or to common cytotoxic T cell epitopes in the vaccine and challenge HA, may contribute to or be responsible for the cross-clade protection. Antibody-dependent cellular cytotoxicity was detected in humans in the absence of neutralization to various strains, including a H5N1 HA [12]. New approaches using recombinant technologies to identify universal influenza vaccines that improve the level of cross-protection are being investigated [15].

The limitations of our study include the lack of investigations on the mechanism of protection in the presence of low or undetectable levels of anti-HA antibodies against the challenge virus. Moreover, some wild-type H5N1 isolates may be more pathogenic in quails than the A/Swan/Nagybaracska/01/06(H5N1) challenge virus, hence requiring higher levels of immune responses for protection. Aspects of these limitations demand further studies.

In summary, we report that (1) small Chinese painted quails may serve for testing vaccine-induced protection against avian H5N1 virus strains, and (2) a single dose of NIBRG-14 vaccine containing 6 μg HA induces anti-HA and neutralizing antibodies at low titres against the homologous strain in all quails and seroconversion against the heterologous strain in only some of the quails, but it confers 100 % protection in those challenged with high doses of the highly pathogenic heterologous (clade 2) strain A/Swan/Nagybaracska/01/06(H5N1). Thus, the conventionally used anti-HA titer might not be a good indicator for protection against influenza A (H5N1) virus.
